# Deepening of response to prostate-specific membrane antigen ligand-targeted radioligand therapy beyond end of treatment

**DOI:** 10.1007/s00259-021-05335-x

**Published:** 2021-04-02

**Authors:** Thorsten Derlin, Elke Krischke, Tobias L. Ross, Frank M. Bengel

**Affiliations:** Department of Nuclear Medicine, Hannover Medical School, Carl-Neuberg-Str. 1, 30625 Hannover, Germany

Prostate-specific membrane antigen (PSMA)-targeted radioligand therapy (RLT) has demonstrated high anti-tumor activity in advanced-stage, metastatic castration-resistant prostate cancer (mCRPC) [1, 2].

Here, we report continued long-term anti-tumor effects after discontinuation of RLT in an 88-year-old patient with progressing disseminated bone and lymph node metastases (a) following therapy with leuprorelin acetate, abiraterone acetate, docetaxel, and denosumab. He received a total of 4 cycles of [^177^Lu]Lu-PSMA-617 RLT between August 2018 and January 2019 (b). Follow-up PET/CT (c) demonstrated a good partial remission with several PSMA-expressing residual bone metastases, and the PSA was 7.6 μg/L. He then had to discontinue RLT due to denosumab-related osteonecrosis of the jaw requiring extensive surgical treatment. Following a complicated clinical course with prolonged infection, he did not resume RLT, since PSA continued to decline without any tumor-specific treatment apart from continued leuprorelin acetate (August 2019: 0.43 μg/L; February 2020: 0.16 μg/L; August 2020: 0.05 μg/L). Follow-up demonstrated complete remission in PET imaging (d) and further PSA decline to the detection limit (February 2021: 0.03 μg/L).

A small subset of patients may achieve long-term complete remission after both ^225^Ac- and ^177^Lu-labeled PSMA-617 if having undetectable PSA after the last RLT [2–4]. Usually, however, patients eventually show PSA progression [2]. The observed continuously deepening response after discontinuation of RLT provides a rationale for further research exploring the optimal duration of PSMA-targeted RLT and predictors identifying candidates for treatment de-intensification or termination. Immune mechanisms similar to using radiation therapy to augment responses to immunotherapy via the abscopal effect in mCRPC may be involved [5].


